# Hyperhomocysteinemia disrupts retinal pigment epithelial structure and function with features of age-related macular degeneration

**DOI:** 10.18632/oncotarget.7384

**Published:** 2016-02-14

**Authors:** Ahmed S. Ibrahim, Suchreet Mander, Khaled A. Hussein, Nehal M. Elsherbiny, Sylvia B. Smith, Mohamed Al-Shabrawey, Amany Tawfik

**Affiliations:** ^1^ Department of Oral Biology and Anatomy, College of Dental Medicine, Augusta University, Augusta, GA, USA; ^2^ James and Jean Culver Vision Discovery Institute, Medical College of Georgia (MCG), Augusta University, Augusta, GA, USA; ^3^ Department of Biochemistry and Clinical Biochemistry, Faculty of Pharmacy, Mansoura University, Mansoura, Egypt; ^4^ Oral and Dental Research Division, Department of Surgery and Medicine, National Research Center, Cairo, Egypt; ^5^ Department of Cellular Biology and Anatomy, MCG, Augusta University, Augusta, GA, USA; ^6^ Department of Ophthalmology, MCG, Augusta University, Augusta, GA, USA

**Keywords:** age related macular degeneration, hyperhomocysteinemia, retinal pigment epithelium, cystathionine-β-synthase and mouse, Gerotarget

## Abstract

The disruption of retinal pigment epithelial (RPE) function and the degeneration of photoreceptors are cardinal features of age related macular degeneration (AMD); however there are still gaps in our understanding of underlying biological processes. Excess homocysteine (Hcy) has been reported to be elevated in plasma of patients with AMD. This study aimed to evaluate the direct effect of hyperhomocysteinemia (HHcy) on structure and function of RPE. Initial studies in a mouse model of HHcy, in which cystathionine-β-synthase (*cbs*) was deficient, revealed abnormal RPE cell morphology with features similar to that of AMD upon optical coherence tomography (OCT), fluorescein angiography (FA), histological, and electron microscopic examinations. These features include atrophy, vacuolization, hypopigmentation, thickened basal laminar membrane, hyporeflective lucency, choroidal neovascularization (CNV), and disturbed RPE–photoreceptor relationship. Furthermore, intravitreal injection of Hcy *per se* in normal wild type (WT) mice resulted in diffuse hyper-fluorescence, albumin leakage, and CNV in the area of RPE. *In vitro* experiments on ARPE-19 showed that Hcy dose-dependently reduced tight junction protein expression, increased FITC dextran leakage, decreased transcellular electrical resistance, and impaired phagocytic activity. Collectively, our results demonstrated unreported effects of excess Hcy levels on RPE structure and function that lead to the development of AMD-like features.

## INTRODUCTION

Age-related macular degeneration (AMD) is the most common and serious sight-threatening complication in people over age 60 [1]. The clinical progression of AMD is categorized in stages according to changes in the macula. It starts with thinning of macular tissues that is not clinically evident, accompanied by obvious depositing of extracellular debris underneath the retina that can be seen upon ophthalmoscopic examination as yellow spots known as drusen. This early stage of macular degeneration is known as dry AMD. The advanced form of dry AMD is called geographic atrophy (GA), in which cells in the epithelial lining of the retina start to degenerate, resulting in regional loss of the retinal pigment epithelial cells (RPE), followed by death of the overlying photoreceptors [2]. Many patients with GA may progress to wet AMD, which is characterized by growth of abnormal blood vessels from the choroid through Bruch's membrane under the macula in a process called choroidal neovascularization (CNV). At this stage, the newly formed blood vessels are fragile, allowing the blood to leak out of the vessels, resulting in hemorrhage, damage the macula, and blindness [3].

In the USA, approximately 1.75 million persons have advanced AMD with associated vision loss and this number is expected to grow to almost 3 million by 2020 [4]. As such, AMD is a widespread public health problem which has considerable impact on the patient's quality of life, the health care system, and society. Current therapeutic strategies including implantable telescope, anti-VEGF therapies, photodynamic therapy, and laser photocoagulation are limited by their invasiveness, cost, and risk of complications. Therefore, it is worthwhile to explore new therapeutic avenues to improve AMD by unraveling its pathophysiology.

In recent years, human studies have highlighted the strong association between hyperhomocysteinemia (HHcy) and the development of AMD [5]. Homocysteine (Hcy) is a key intermediate in the metabolism of methionine and is converted by cystathionine β-synthase *(cbs)* to cysteine (transsulfuration pathway) or by methionine synthase back to methionine (remethylation pathway). These enzymatic reactions are dependent on adequate supply of vitamins B_12_, folate, and pyridoxine. Disturbance in these pathways results in the accumulation of Hcy and its active metabolite Hcy-thiolactone in the blood, which are independent risk factor for neurodegenerative and cardiovascular diseases. In a clinic-based study of 60 patients, elevated plasma Hcy and lower vitamin B_12_ concentrations were associated with the prevalence of AMD [6]. Moreover, cross-sectional data collected from the Blue Mountains Eye Study of 2335 participants reported that the elevated serum Hcy predicts increased risk of incident AMD [7]. Results from population-based studies support the notion that increased serum total Hcy is independently associated with increased odds of AMD [8]. Thus far, there is no definitive experimental evidence a direct cause-effect relationship between Hcy and retinal RPE dysfunction, which plays a fundamental role in the development and progression of AMD. Previous studies using a mouse with excess levels of homocysteine, due to lack/deficiency of the enzyme cystathionine-β-synthase (*cbs^−/−^ and cbs^+/−^* mice), reported histological including RPE alterations [9] as well as functional (ERG) changes [10].

The current study was undertaken to investigate whether elevated Hcy acts directly on RPE structure and function. Furthermore, the present work aimed to gain insights in the molecular mechanisms involved by validating the causal relationship between increased Hcy and disorganization of tight junction proteins, an indicator of altered barrier function of RPE. To accomplish these aims, an *in vivo* mouse model of excess Hcy (due to *cbs* deficiency) and *in vitro* human retinal pigment epithelial cell line (ARPE-19) model were used.

## RESULTS

### Optical coherence tomography (OCT) and fluorescein angiography (FA) examinations

**Figure 1:**
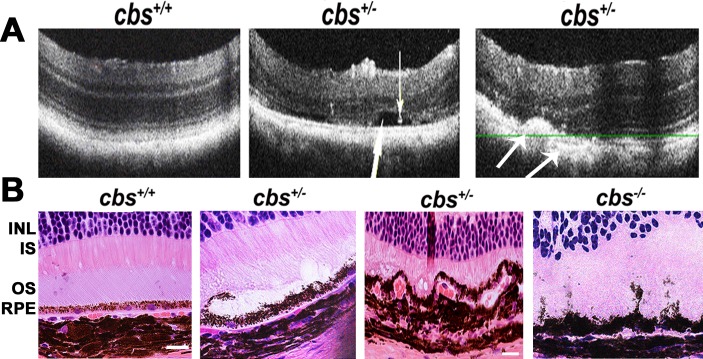
Optical Coherence Tomography (OCT) and histological assessment **A.** OCT examination of *cbs^+/+^* and *cbs^+/−^* mice (24weeks). b-scans of *cbs^+/+^* mouse (left panel) and *cbs^+/−^* mouse (middle and right panels). The *cbs^+/−^* retina (middle panel) showed separation (big white arrow) and precipitation (small white arrow) at the RPE-photoreceptor interface, while the *cbs^+/−^* retina (right panel) showed thickening at the area of the choroidal blood vessel and decreased the thickness of the photoreceptor layer with accumulations of opaque structureless material with hyporeflective subretinal lucency (medium size arrows) at the RPE-choroid interface suggesting RPE-choroidal abnormality (*n* = 8). **B.** Histological assessment of cbs*^+/+^, cbs^−/−^* and *cbs^+/−^* mice with edema, vaculation, irregularity and degeneration of the RPE in the *cbs^+/−^* and *cbs^−/−^* retinas (Calibration bar is 50 μm and *n* = 9).

**Figure 2:**
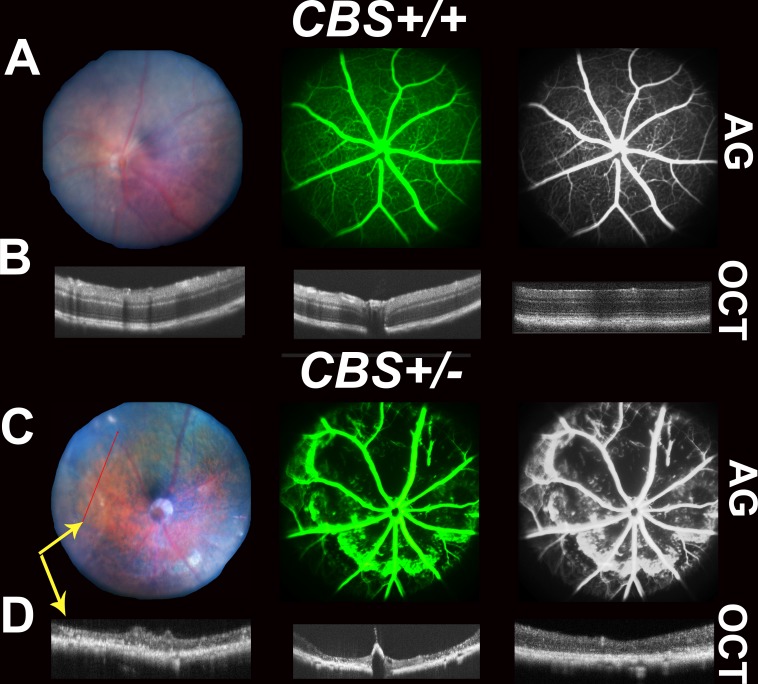
Retinal Fundus examination, Fluorescein angiogram (FA) and Optical Coherence Tomography (OCT) assessment of 64-week *cbs*^+/+^ and *cbs*^+/−^ mice Fundus examination, FA and OCT were performed on *cbs^+/+^* and *cbs^+/−^* mice (64 week). **A.** and **C.** Representative fundus and angiograms for *cbs^+/+^* and *cbs^+/−^*, showing normal fundus with well-formed vessels in *cbs^+/+^* mice; however, fundus and angiograms for *cbs^+/−^* mouse retinas exhibit apparent geographic atrophy reflecting retinal loss with severe decrease in the vasculature with diffused hyperfluorescence. The most right panels are black and white images processed using the Adobe Photoshop to permit visualization of vessels. **B.** and **D.** OCT examination showing normal appearance of retina in the *cbs^+/+^* mice, but reminiscent of geographic atrophy/dry AMD with marked reduction in retina thickness and focal hyper reflective spots in *cbs*^+/−^ mice, (*n* = 5 mice). Yellow arrows (C&D) are indicating that images were taken by both FA and OCT simultaneously.

**Figure 3:**
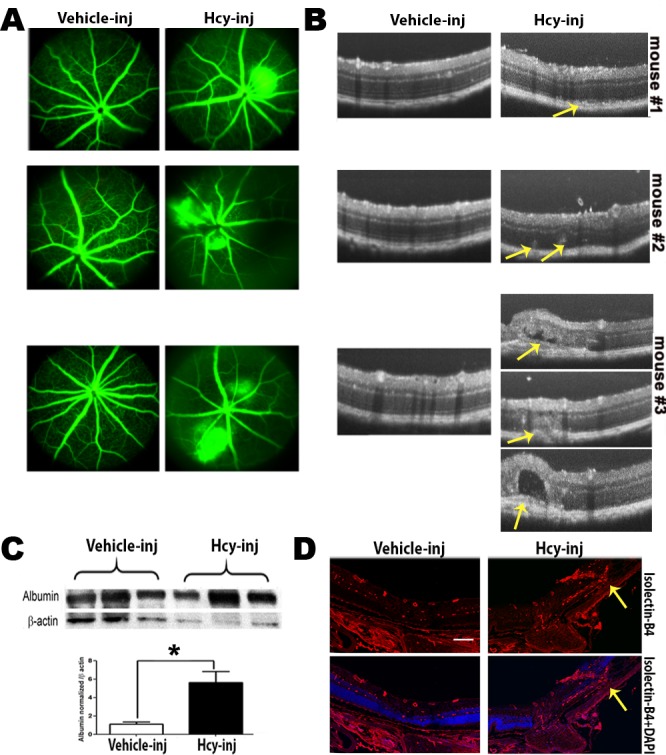
Evaluation of Retinas of C57BL6 mice injected intravitreally with Hcy-thiolactone **A.** Fluorescein angiogram evaluation, showing fluorescein leakage with focal areas of hyperfluorescence in Hcy-injected mice compared to normal retinal appearance in vehicle-injected eyes. The leakage was confirmed by measuring the albumin leakage in the retinas by western blotting, which was significantly increased in the Hcy-injected mice eyes, *p < 0.05 **C. B.** OCT examination revealed features of AMD, such as hyporeflective subretinal lucency (mouse 1&2, yellow arrows), sub retinal fluid and choroidal neovascularization (mouse 3, yellow arrows). **D.** Choroidal neovascularization were confirmed by staining of the frozen section with blood vessel marker isolectin-B4 (yellow arrows).

*OCT evaluation* of the retina in 24 weeks living *cbs^+/+^* and *cbs^+/−^* mice showed a normal appearance in *cbs*^+/+^ mice, while, retinas of *cbs^+/−^* showed an uneven appearance and changes similar to those occur in AMD. These features include decreased retinal thickness, atrophy at the level of the photoreceptors, subretinal fluid accumulation, hyporeflective subretinal lucency, thickened basal laminar membrane, separation of RPE, and increased thickness in the area of the choroid suggesting a possible choroidal neovascularization (CNV) (Figure 1A). Additionally, OCT examination of older mice (64 week) revealed reminiscent of geographic atrophy/dry AMD with a marked reduction in retina thickness and focal hyper reflective spots (Figure 2D), compared to normal OCT appearance in the age matched *cbs^+/+^* mice (Figure 2B). These reflective spots are suggestive of RPE migration and/or inflammatory cell infiltration. Furthermore, OCT examination of mice injected intravitreally with Hcy (Figure 3B) showed features of AMD, such as hyporeflective subretinal lucency (mouse 1&2, yellow arrows), subretinal fluid, and CNV (mouse 3, yellow arrows), compared to vehicle injected controls. CNV formation was confirmed in retinal frozen sections by staining with the blood vessel marker, isolectin-B4 (Figure 3D, yellow arrow).

*FA examination* of 64 week-old *cbs*^+/+^ mice showed normal fundus (Figure 2A), while FA examination of cbs^+/−^ mice (Figure 2C) revealed a fundus with apparent geographic atrophy reflected by retinal loss with a sever decrease in retinal vasculature coupled with a diffuse hyper fluorescence, a suggestive of RPE atrophy. The FA from the Hcy-injected mice eyes (Figure 3A) showed diffuse hyper fluorescence and leakage as well as focal areas of hyper-fluorescence coming from the deeper layer (mouse #3) suggestive of choroidal neovascularization. Fluorescein hyper-fluorescence was confirmed to be due to vascular leakage by measuring the albumin leakage from the blood vessels into the retina after perfusion, which was significantly higher in the Hcy injected eyes *versus* the vehicle injected controls (Figure 3C).

### Histological assessment

Histological assessment of *cbs^+/+^* *, cbs^+/−^* and *cbs^−/−^* mice (examined at age of 5 weeks due to the fact that the *cbs^−/−^* mice have a short lifespan ranging ∼ 3-5 weeks) has been performed. The *cbs^+/+^* retina showed normal histologic appearance with healthy RPE and normal RPE- photoreceptor interaction, while retinas of the *cbs*^−/−^ mice revealed abnormal RPE morphology with increased pigmentation, vacuoles, edema, subretinal fluid, separation and disrupted relation with the photoreceptors (Figure 1B). Interestingly histological examination of the retinas from 30 weeks *cbs*^+/−^ mice confirmed the OCT data and showed features similar to AMD, such as disrupted RPE- photoreceptor relation with edema, accumulation of subretinal fluid, and abnormal looking RPE as well as choroidal blood vessels. Interestingly, our finding is confirming the pervious observation of RPE alteration in the *cbs^−/−^* when retinas were examined at light and EM levels [9].

### Electron microscopy evaluation

**Figure 4:**
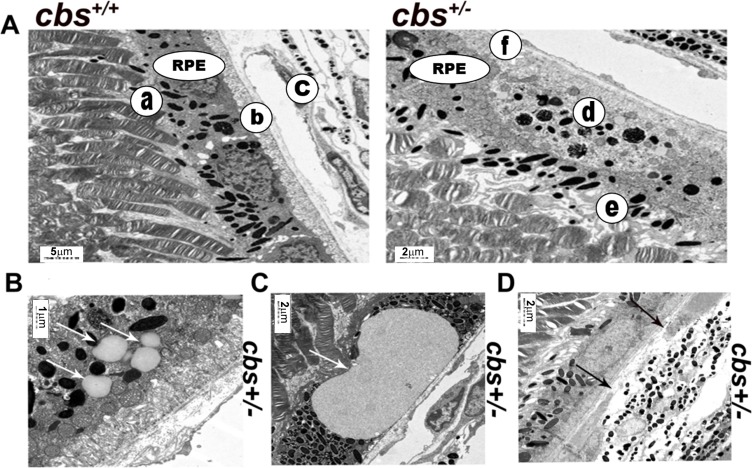
Electron microscopy evaluation of *cbs*^+/−^ retinal RPE (6-8wks) **A.** Ultra-structural evaluation of the *cbs^+/+^* mice revealed normal looking RPE (left panel) with cuboidal RPE cells which have very long sheet-like apical microvilli that project and interact with the photoreceptor outer segments (a) and highly folded basal surface resting on Bruch's membrane (b) separating the RPE from the choriocapillaries (c). In contrast, *cbs^+/−^* mouse retina (right panel) revealed abnormal looking RPE with less pigmentation and accumulation of the pigmented particles in the lower part of the RPE instead of the apical part (d), lost apical microvilli and disturbed RPE -photoreceptor relation (e) and thickened Bruch's membrane (f). **B.** and **C.** showing structureless material between RPE and bruch's membrane (white arrows). **D.** Interruption of the bruch's membrane underneath the RPE (black arrows). (Calibration bar; 5 μm, 2 μm, 1 μm, 2 μm and 2 μm) and *n* = 6.

Next, we investigated the ultrastructural morphology of the RPE cells in *cbs^+/+^* and *cbs^+/−^* mouse retinas using electron microscopy (Figure 4). *cbs^+/+^* mouse retina (Figure 4A, left panel) revealed normal cuboidal RPE cells with very long sheet-like apical microvilli that project and interact with the photoreceptor outer segments (a) and highly folded basal surface which interact with the underlying Bruch's membrane (b) separating the RPE from the choriocapillaries (c). In contrast, the *cbs^+/−^* mouse retina (Figure 4A, right panel) showed abnormal RPE structure with less pigmentation and accumulation of pigmented particles in the lower part of RPE instead of the apical part (d), lost apical microvilli and disturbed RPE -photoreceptor relation (e) as well as thickened Bruch's membrane (f). Furthermore, the EM exanimation of the *cbs^+/−^* mouse retinas showed accumulation of extracellular material between Bruch's membrane and the RPE (Figure 4B and 4C) suggesting a possible drusen formation. Disruption of the Bruch's membrane was also observed in (Figure 4D).

### Examination of outer blood-retinal barrier junction proteins

#### A) *In vivo*

The aforementioned data showing alteration in RPE cell morphology in retinas of *cbs^+/−^* mice, prompted us to investigate the barrier function of RPE by evaluating the markers of intact outer blood-retinal barrier (BRB). Tight junction proteins (TJPs); ZO-1 and occludin were investigated by immunofluorescence in RPE flat-mounts from *cbs^+/+^* and *cbs^+/−^* mice (24-30 weeks) as shown in Figure 4A. A uniform pattern of typical hexagonal morphology of the RPE recognized the expression of ZO-1 (green) and occludin (red) between RPE cells were observed in the RPE flat-mounts from the *cbs^+/+^* mouse retinas, while disrupted, interrupted and attenuated ZO-1 and occludin expressions were observed in the retinas of the *cbs^+/−^* mice indicating disrupted outer BRB in the retinas of hyperhomocysteinemic mice.

#### B) *In vitro*

**1. Evaluation of tight junction proteins and cytoskeletal microfilament**

The alteration we observed in RPE structure of *cbs*^−/−^ and *cbs*^+/−^ mice and the changes in the TJPs in the RPE flatmounts of the *cbs*^+/−^ mice led us to study the effect of excess Hcy on the ARPE-19 cell barrier function (Figure 6). The formation of intact blood-retinal barrier (BRB) by the RPE is mainly reliant on the function of tight junctions to create a controlled diffusion barrier to non-transported solutes [11]. As shown in (Figure 6A), continuous ZO-1 and occludin was observed between RPE cells in the control, while attenuated, interrupted, and diminished ZO-1 and occludin was observed between RPE cells treated with Hcy (50 μM) for 18-24 hours. TJPs protein expression was confirmed with western blot analysis (Figure 6B and 6C), showing that Hcy (50 μM) decreased TJPs expression, which decreased more when Hcy concentration increased to 100 μM. Interestingly, rapid loss of occludin was more evident than the loss of ZO-1. The decreased levels of the two TJPs suggest compromised integrity of the outer BRB. In addition, we checked the expression of F-actin and its relation to ZO-1 in ARPE-19 under Hcy treatment (Figure 6D; upper panel low magnification and lower panel, high magnification), we noticed normal and continuous expression of both ZO-1 and F-actin in non-treated RPE cells, while, interrupted F-actin expression in the Hcy-treated cell. Interestingly the areas where F-actin was disturbed also showed attenuated and interrupted ZO-1 expression. These data presumably reflect a tight junction and cytoskeleton disorganization in Hcy-mediated RPE dysfunction and reflect the importance of the interaction between the TJPs and cytoskeletal microfilament to maintain the polarized structure of RPE.

**Figure 5:**
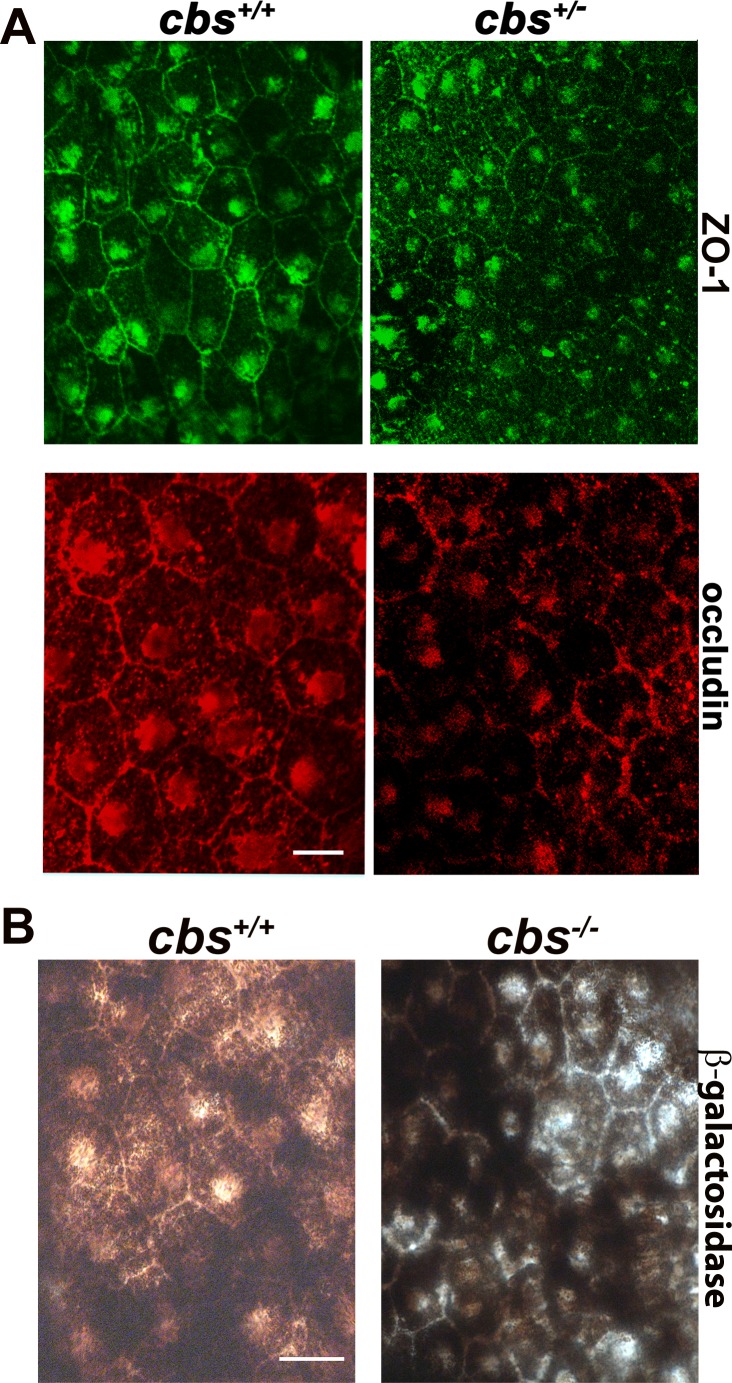
Evaluation of Outer Blood-Retinal Barrier **A.** RPE flat-mounts from the *cbs^+/+^* and *cbs^+/−^* mice (24-30wks) immunostained for markers of tight junction proteins; ZO-1 (green) and occludin (red). **B.** β-galactosidase activity evaluation, as a biomarker for senescence in RPE flat-mounts. Calibration bar is 10 μm and *n* = 14.

**Figure 6:**
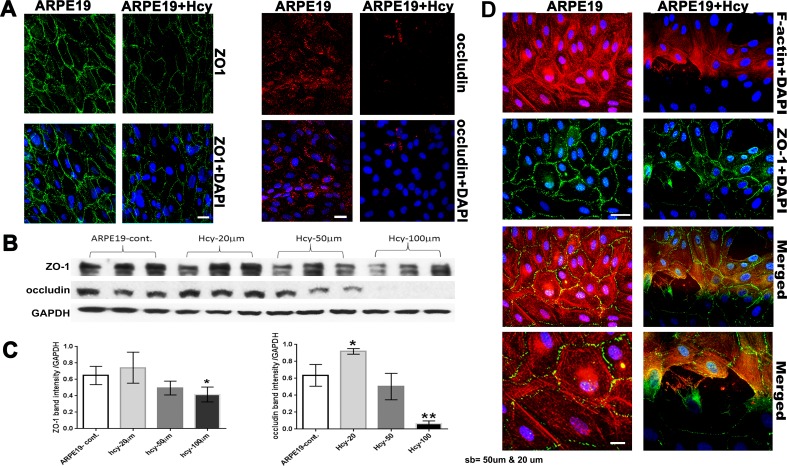
Evaluation of Tight junction proteins and cytoskeletal microfilament of ARPE-19 cells treated with and without Hcy-thiolactone **A.** Immunofluorescence expression of ZO-1 and occludin. Calibration bar: 50 μm. **B.** Western blot analysis of ZO-1 (198 kD) and occludin (59 kD), GAPDH was used as the loading control. **C.** Quantification of densitometric scans of protein bands showing significant decrease in occludin and ZO-1 expression in the ARPE19 cells treated with 50 μM Hcy-thiolactone. **p < 0.01. **D.** Evaluation of the stress fibers (F-actin) by Immunofluorescence (upper panel; lower magnification) and (lower panel; higher magnification). Calibration bar; 50 μm and 10 μm, respectively.

**2. Transendothelial electric resistance (TER) and FITC dextran flux assay**

**Figure 7:**
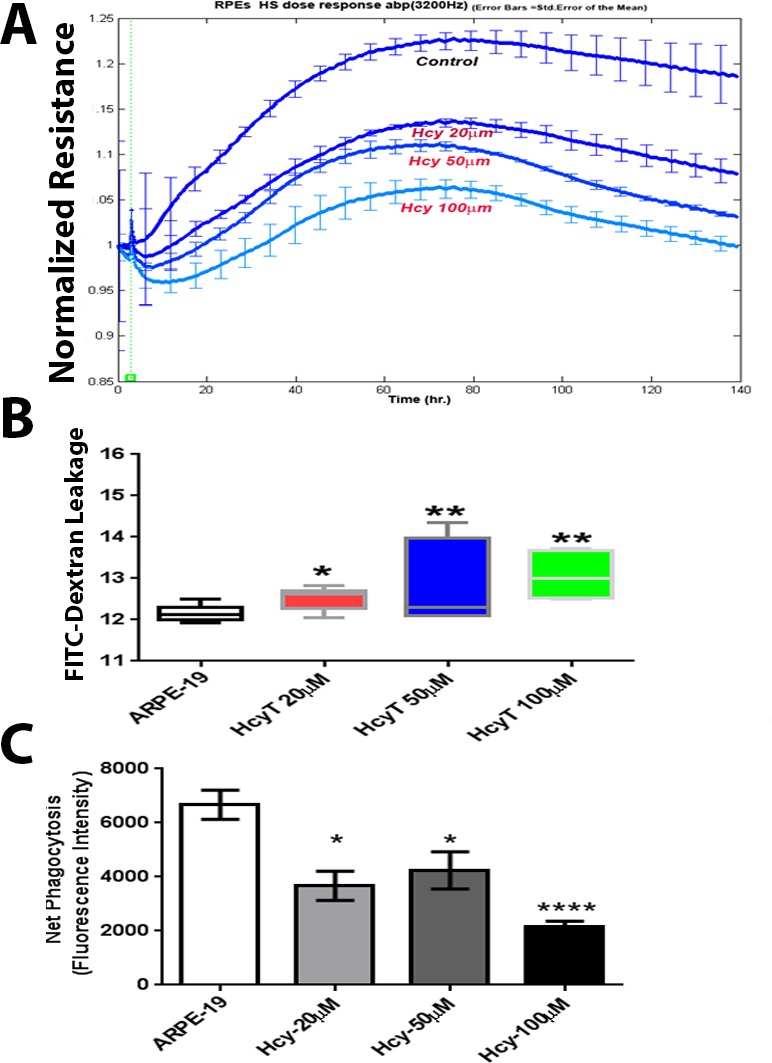
Transendothelial electric resistance (TER), FITC dextran flux, and phagocytic activity assays of ARPE-19 cells treated with and without Hcy-thiolactone **A.** TER, which revealed significant decrease in TER in the Hcy-treated cells, **B.** FITC dextran flux through the confluent monolayer, which revealed significant increase in FITC dextran leakage in hcy-treated cells. **C.** Evaluation of RPE cells phagocytic function of ARPE19 cells treated with and without different concentration of Hcy-thiolactone, which demonstrated a significant decrease in Hcy-treated RPE cells *p < 0.05, **p < 0.01 and ***p < 0.001.

We next performed functional assays and investigated whether elevated Hcy would disrupt barrier function in ARPE-19 monolayer using real-time analysis of transendothelial electric resistance (TER), an indicator of monolayer integrity. Cells treated with different concentrations of Hcy (20, 50, or 100 μM) showed significantly reduced TER in a dose dependent manner compared to the vehicle-treated pigment epithelial monolayer (Figure 7A). To further confirm the role of excess Hcy in altering the barrier function of epithelial cell layer, we investigated whether Hcy induces permeability changes to FITC dextran flux through the confluent monolayer. ARPE-19 treated with Hcy was significantly more permeable to FITC-dextran compared to controls as indicated by increasing diffusive flux (Po) for FITC-dextran (Figure 7B).

### Evaluation of senescence in *cbs*^+/−^ mice

β-galactosidase activity was evaluated in RPE flatmount preparations from *cbs^+/+^*and *cbs^+/−^* mice (24weeks). β-galactosidase activity, a commonly used biomarker to evaluate replicative senescence in mammalian cells [12], was analyzed by immunohistochemistry staining. As shown in Figure 5B, RPE senescence-associated β-galactosidase activity was detected in *cbs^+/−^* mouse RPE cells when compared with *cbs^+/+^* mice.

### Evaluation of RPE cells phagocytic function

In addition to their role in maintaining the outer retinal barrier, RPE cells also play an important role in the daily phagocytosis of the photoreceptor outer segments, which is essential for vision. RPE cells use an uptake machinery that is very similar to the one microphages use to phagocytose apoptotic cells [13]. F-actin assembly is essential for the uptake machinery of RPE cells. Our data revealed disassembly of F-actin in Hcy-treated RPE cells; therefore we evaluated the RPE phagocytic activity with different concentrations of Hcy. Hcy-treated RPE exhibited significantly reduced phagocytic capacity to engulf apoptotic particles compared to vehicle-treated control (Figure 7C).

## DISCUSSION

AMD is characterized by photoreceptor loss associated with reduced central vision. Recent studies have recognized RPE dysfunction as a potential culprit mechanism contributing to photoreceptor cell death in the context of AMD [14-16]. However, the molecular mechanism by which age-related RPE dysfunction occurs remains a critical missing link. If such a link is established, clinical interventions with agents that target these early features of AMD would provide long-term visual benefits. In the current study, we put forward a new concept of impaired homocysteine metabolism in the genesis of abnormal RPE structure and function with AMD-like features.

RPE is a polarized tight-cell monolayer that functions to control the maintenance and homeostasis of retinal photoreceptors [17]. The apical ends of these cells engulf outer segment shed from photoreceptors by phagocytosis, which is profoundly essential to the survival of photoreceptors [18]. The basolateral side of RPE transports nutrients from the choroidal circulation to photoreceptors, meanwhile, it provides outer blood-retinal barrier that blocks nonspecific diffusion from the choroid. RPE cells also regenerate 11-cis retinal and move it back to photoreceptors to maintain the visual cycle. Failure of one or more of these essential RPE cell functions brings about the progression of many retinal degenerative diseases such as AMD. This input has originated partly from studies on whole mount preparations that showed RPE density decreased with age [19] followed by collateral loss of neighboring photoreceptor cells and choriocapillaries [20]. These initial observations have been supported in postmortem AMD human retinas showing a statistically significant increase in TUNEL-positive RPE cells compared with the control ones [21] and reinforced experimentally by showing that RPE cell grafts prevented photoreceptor degeneration in Royal College of Surgeons rats [22]. Furthermore, the *in vitro* studies on co-cultured retinal neurons seeded over ARPE-19 cells have shown that photoreceptors attached to the apical surfaces of RPE and proceeded with their normal development, indicated by the increased synthesis of rhodopsin [23]. All of this seemingly overwhelming evidence has sculpted the concept of dysfunctioned RPE as a contributor factor in AMD. In concordance with these previous studies, we observed similar changes in morphology and barrier function of RPE under hyperhomocysteinemic conditions, such as RPE atrophy, vacuolization, hypopigmentation, and disturbed RPE-photoreceptor relationship accompanied by a corresponding decrease in the expression of multiple TJPs and possible choroidal neovascularization. However, this study is the first to report these changes in a mouse model of hyperhomocysteinemia demonstrating the development of RPE degeneration in the retina with certain phenotype features resembling AMD.

Although it has been reported that Hcy level was elevated in patients with AMD [5], the mechanisms underlying hyperhomocysteinemia in AMD remain unclear. Major determinants are genetic mutations of Hcy metabolizing enzymes as well as nutritional deficiencies in circulating folate, vitamin B_12_, and vitamin B_6_, which are required for regulating the activity of *cbs*. *Cbs* converts Hcy to cysteine *via* the transsulfuration pathway leading to the formation of three important products: glutathione, a major antioxidant; hydrogen sulfide, a signaling molecule that has a role in neurological homeostasis [24]; and taurine, the most abundant amino acid in retina. Given these facts, an intriguing question has been raised as whether is it Hcy *per se* that leads to the observed RPE damage in *cbs*^+/−^ mice or the decreased levels of cysteine and its downstream metabolites?. Accordingly, two complementary approaches have been conducted to analyze the direct effect of Hcy on RPE. The first approach was to inject Hcy locally into the mouse eye and the second approach was to treat human retinal pigment epithelial monolayer *in vitro* with Hcy. Injection of Hcy induced many characteristic features of AMD, including hyporeflective lucency and choroidal CNV. Our *in vitro* studies demonstrated notable findings: First, Hcy produced a dose- and time-dependent increase in RPE monolayer permeability associated with a decrease in the expression of the TJPs ZO-1 and occludin. Second, Hcy dose-dependently and significantly impaired RPE phagocytic activity, another prominent feature associated with age-related RPE dysfunction. Daily phagocytosis of photoreceptor outer segment disks by RPE uptake machinery is essential for vision and requires F-actin assembly. To gain insight into the underlying mechanisms, we broadened the scope of this study to determined whether the decreased RPE phagocytosis accompanying Hcy-treatment, would be associated with disassembly of F-actin. Our data demonstrate that F-actin was disassembled and aggregated in Hcy-treated RPE cells.

Preservation of the outer BRB is essential for retinal homeostasis. The outer BRB is formed by TJPs between RPE cells that rest upon Bruch's membrane separating neural retina from the choriocapillaries. TJPs facilitate cell adhesion and are essential to the BRB because they control paracellular diffusion across epithelia [25]. In addition, they have a role in signaling pathways involved in epithelial differentiation, proliferation and gene expression [26]. While this study represents the first systematic investigation of outer BRB in the *cbs^+/−^* mutant mouse, there are a number of reports of severe vasculopathy in other tissues of this mutant mouse model. For example, our previous studies reported an alteration of the inner BRB vasculature, including ischemia concomitant with neovascularization and decreased expression of TJPs in the retinal vessels of *cbs*^−/−^ or cbs^+/−^ mice [27,28]. TJPs are important components in the inner BRB formed between retinal endothelial cells, which rest on a basal lamina covered by foot processes of astrocytes and Müller cells.

Several mechanisms have been proposed regarding how elevated Hcy impairs barrier function that can be applied to RPE cells dysfunctions seen in our model. One such mechanism refers to the synthesis of a reactive intermediate known as thiolactone Hcy that leads to protein damage by a mechanism involving homocysteinylation of protein lysine residues [29]. The extent of thiolactone synthesis depends on relative level of Hcy, the activities of methionine synthase and *cbs* [30], and their cofactors such as vitamin B_12_, folate and vitamin B_6_. When the ratio of intracellular Hcy to methionine is high, the enzyme that conjugates methionine to its t-RNA ‘methionyl-tRNA synthase (MetRS)’ will utilize Hcy instead of methionine, causing the thiol group and carboxyl group of Hcy to join with each other by thioester linkage resulting in the generation of cyclic derivative Hcy-thiolactone [31]. Moreover, mutation in genes encoding methionine synthase and *cbs* leads to enhanced metabolic conversion of Hcy to the thiolactone [30]. The thioester group of thiolactone combines with any lysine residues but not histidine and arginine residues in proteins in a process known as N-homocysteinylation [32]. This incorporation of thiolactone into functional protein alters protein conformation and subsequently its biological activity [33]. In occludin, there is a cluster of conserved lysine residues that contribute to its stability and its complex with ZO-1 [34]. The direct homocysteinylation of these lysine residues may explain why apical tips of the RPE's were prone to gross structural changes upon the treatment with Hcy-thiolactone. Besides Hcy's ability to homocysteinylate many proteins, other reported effects might have contributed to our finding of the deleterious effect Hcy on RPE, such as the induction of oxidative stress involving NADPH oxidase, thioredoxin, and protease-activated receptors [35]. Alterations in the expression of multiple genes induced by Hcy [36] could also contribute to RPE's dysfunction in *cbs* mice model.

Collectively, the experiments in this study provide new insights in understanding the pathogenesis of AMD, demonstrating that impairment of *cbs* activity and the accumulation of Hcy within the retina alter RPE structure and functions. Hcy significantly attenuated the expression and organization of RPE's tight junction proteins both *in vitro* and *in vivo*. The current study shows the deleterious effects of Hcy on RPE and predicts the future clinical amalgamation of Hcy-clearing mechanisms with existing and promising treatments for AMD.

## MATERIALS AND METHODS

### Animals

All procedures with animals conformed to the ARVO Statement for Use of Animals in Ophthalmic and Vision Research and were performed in accordance with the Public Health Service Guide for the Care and Use of Laboratory Animals (Department of Health, Education, and Welfare publication, NIH 80-23) and Augusta University guidelines. The generation of mice deficient in cystathionine beta-synthase (*cbs*) has been described by Watanabe et al [37]. Breeding pairs of *cbs^+/−^* mice (B6.129P2-Cbs^tm1Unc^/J; Jackson Laboratories, Bar Harbor, ME) were used to establish colonies of *cbs^+/+^, cbs^+/−^*, and *cbs^−/−^* mice. All animals were maintained in clear plastic cages, subjected to standard 12-hour light/12-hour dark light cycles in the animal room at regulated temperature (22 to 24°C), and allowed to eat and drink *ad libitum*. Light levels at the bottom of cages were controlled at 1.5-foot candles (16.1 lux) to avoid the possibility of light damage to the retina. Mice were genotyped according to the Jackson animal laboratory's protocol. For RPE evaluation, mice were used at ages ranging from 5-64 weeks.

For intravitreal injection, the procedure was the same as previously described [38]. To avoid uncontrolled intraocular pressure increase, the volume of intravitreal injections was limited to 1 μL. L-Homocysteine thiolactone hydrochloride (Sigma-Aldrich, Louis, MO) was dissolved in water and a working solution of 10x was prepared by diluting 1 μL of stock solution (200 mM) to 100 μL with PBS, assuming the vitreous volume of mouse eye is ∼10 μL [39]. Then by injecting 1 μL of this working solution, 200 μM vitreal concentration of Hcy-thiolactone was obtained. The volume of the injected solution apparently did not cause significant pressure-induced retinal damage, because PBS-injected control eyes showed normal retinal morphology with no apparent apoptosis within 7 days. The dose of Hcy-thiolactone was chosen according to what has been previously published (200 μM) [40].

### Optical coherence tomography (OCT) and Fluorescein Angiography (FA)

To evaluate retinal RPE structure and function *in vivo*, OCT and FA were performed simultaneously as described previously with some modifications [41]. Briefly, mice were anesthetized using 2% isoflurane and their pupils were dilated using 1% tropicamide eye drop. Each mouse was then placed on the imaging platform of the Phoenix Micron III retinal imaging microscope supplemented with OCT imaging device (Phoenix Research Laboratories, Pleasanton, CA). Genteal gel was applied liberally to keep the eye moist during imaging. Mice were administered 10 to 20 μL 10% fluorescein sodium (Apollo Ophthalmics, Newport Beach, CA), and rapid acquisition of fluorescent images ensued for ∼5 minutes. Fluorescein leakage manifests as indistinct vascular borders progressing to diffusely hazy fluorescence.

### Electron microscopy

Mice were perfused with a 2% paraformaldehyde/2% glutaraldehyde in 0.1 M sodium cacodylate buffer solution. Thereafter, eyes were enucleated and fixed for 1 hour at room temperature in 2% paraformaldehyde/2% glutaraldehyde in 0.1 M sodium cacodylate buffer in sucrose (pH 7.2) and postfixed for 1 hour with osmium tetroxide. Eyes then were prepared for EM examination per our method [27]. Briefly, eyes were punctured at the limbus, fixed overnight, and washed in sodium cacodylate buffer. Then they were dehydrated in serial ethanol (70%-100%), infiltrated with propylene oxide, and infiltrated with Epon and propylene oxide for 1 hour. Subsequently, fresh Epon was added, and the polymerization was carried out overnight. Tissue was embedded in an embedding resin mixture. Thin sections (90 nm) were cut with a diamond knife on an ultramicrotome and placed on copper grids. Sections were stained with uranyl acetate and lead citrate and viewed by a transmission electron microscope (JEM 1230; JEOL USA, Inc., Peabody, MA) supplemented with a CCD camera (UltraScan4000/First Light Digital Camera Controller; Gatan Inc., Pleasanton, CA) for Imaging.

### RPE flatmount

Eyes were enucleated, fixed in 4% paraformaldehyde overnight and transferred to PBS. RPE layer was dissected, washed with PBS, and incubated with Power Block (BioGenex, San Ramon, CA, USA). The RPE was then incubated overnight at 4°C with antibodies for tight junction proteins; ZO-1 and occludin, followed by incubation with the appropriate secondary antibodies for 1 hour at 37°C. Additionally, RPE flatmounts were visualized for senescence using a detection kit (Bio vision, K320-250, Milpitas, CA) per manufacturer's instructions. Axioplan-2 fluorescent microscope (Carl Zeiss, Göttingen, Germany) equipped with a high resolution microscope (HRM) camera (Carl Zeiss) has been used to visualize RPE flat mounts. Images were captured and processed using Zeiss Axiovision digital image processing software (version 4.8; Carl Zeiss).

### Human retinal pigmented epithelial cell line (ARPE-19)

ARPE-19 obtained from American Type Culture Collection (ATCC, Manassas, VA, USA). Passages 6-15 of ARPE-19 were grown on gelatin-coated dishes and maintained in Dulbecco's modified Eagle's medium-nutrient mixture F-12 (DMEM/F-12, Thermo Scientific, Wyman, Massachusetts) supplemented with 1% penicillin/streptomycin, and 10% fetal bovine serum (FBS, Atlantic Biological, Norcross, GA, USA).

### Immunofluorescent assessment of tight junction and cytoskeleton and vascular markers

Hcy-treated ARPE cells were fixed in 4% paraformaldehyde for 10 min, washed with PBS, and blocked with Power Block BioGenex, Fremont, CA), Ca. # BS-1310-25 for one hour. Cells were then incubated for 3h at 37°C with anti-ZO1 (Life technology, Cat#: 40-2200402200, diluted at 1/200), anti-occludin (Invitrogen, Cat#: 331500, diluted at 1/200), anti-F-actin (Abcam, ab205, diluted at 1/200) or Biotinylated GSL I - isolectin B4 (Vector Laboratories, Burlingame, Ca), Cat#: B-1205, 7μl/ml). Thereafter, cells were washed 3 times with PBS containing 0.3% Triton-X, incubated with appropriate secondary antibodies (Alexafluor and Texas red avidin, Invitrogen, Eugene, Oregon), and coverslipped with Fluoroshield containing DAPI (Sigma-Aldrich Chemical Corp., St. Louis, MO) as a counter stain. Immunofluorescent signals were detected by confocal microscopy (LSM, Carl Zeiss).

### Protein extraction and western blot analysis

Washed cultured cells as well as retinal tissues were lysed in modified RIPA buffer supplemented with 1:100 (v/v) of proteinase/phosphatase inhibitor cocktail (Thermo Scientific). Insoluble material was removed by centrifugation at 12,000 xg at 4°C for 30 min. Protein was determined by BCA Protein Assay (Thermo Scientific) and equal amount of protein was boiled in Laemmli sample buffer, separated by SDS-PAGE on a gradient gel (4 to 20%, Pierce, Rockford, IL), transferred to nitrocellulose membrane, and incubated with specific antibodies. Antibodies for ZO-1 (Abcam, ab59720), occludin (Invitrogen, 71-1500), Albumin (Bethyl, TX, USA), and GAPDH, (Sigma-Aldrich) were detected with a horseradish peroxidase-conjugated antibody and enhanced chemiluminescence (Thermo Scientific). Intensity of immunoreactivity was measured by densitometry using Image J software (NIH).

### Electric cell-substrate impedance sensing (ECSIS)

Effect of Hcy-thiolactone on barrier function of RPEs was assessed by recording changes in Trans-Cellular Electrical Resistance (TER) using ECIS as previously described [42,43]. Briefly, 96W20idf arrays were used. These arrays were coated with cysteine for 30 minutes then with gelatin for 30 minutes before seeding the ARPE-19 cells at a density of 10^4^/ well in 300 μL full media. Cells were left undisturbed until fully attached forming a confluent monolayer indicated by a capacitance below 10 nF. Cells were then starved for 6 hours in 200 μL serum free media and treated with different concentrations of Hcy-thiolactone (0, 20, 50, or 100 μM). Different treatments were prepared (3X in serum free media) and added to the corresponding wells in 100 μL without removing the existing media covering the confluent monolayer of ARPE-19. The electric currents, passing through the confluent monolayer, were measured and recorded independently in each well by the ECIS system (Applied Biophysic, Inc., Troy, NY.). TER was measured at frequency of 32000 Hertz and recorded over the experimental time course. TER then normalized as the ratio of measured resistance at each time point to baseline resistance and plotted as a function of time.

### Fluorescein isothiocyanate (FITIC)-dextran permeability assay

ARPE-19 cells were seeded on collagen/fibronectin coated membranes with 0.4 μm pores (Transwell; Corning Costar), in normal media. After the cells formed a complete confluent layer, the cells were shifted to serum free media 6 hours and then treated with different concentrations of Hcy (0, 20, 50, or 100 μM) in the apical chambers for 24 hours. Thereafter, FITC-dextran (1 mg/ml, 70 KD, Sigma) was added to the apical chambers followed by obtaining aliquots from the basolateral chamber at 1, 3, and 6 hours, and measuring the fluorescence intensity with a plate reader. The rate of diffusive flux (Po) was calculated by the following formula [44]:

Po = [(FA/Δt)V_A_]/(F_L_A).

Where Po is in centimeters per second; FA is basolateral fluorescence; F_L_ is apical fluorescence; Δt is change in time; A is the surface area of the filter (in square centimeters); and V_A_ is the volume of the basolateral chamber (in cubic centimeters).

### Assessment of phagocytic activity of ARPE-19

ARPE-19 cells were seeded in 96-well plate in full media. After the cells formed a complete confluent layer, the cells were shifted to serum free media for 6 hours and then treated with/without different concentrations of Hcy (20, 50, or 100 μM) for 24 hours. Thereafter, the culture medium was removed and changed to pHrodo red suspension containing pH-sensitive fluorescent phagocytics particles that fluoresce brightly red inside the cells (Invitrogen, Grand Island, NY; 1 mg/mL). The plate was then transferred to 37°C, incubated for 2 hours, and the fluorescence intensity of the engulfed particles was measured by a plate reader at excitation of 550 nm and emission of 585 nm.

### Data analysis

The results are expressed as mean ± SD. Differences among experimental groups were evaluated by using the two-tailed *t* test or one way analysis of variance (ANOVA). When statistical differences were observed using ANOVA, a post hoc Tukey's test was performed to determine which groups differed. A confidence level of *P* < 0.05 was considered statistically significant.
